# Cascara Kombucha: The Role of Fermentation and Particle Size in Enhancing Antioxidant and Bioactive Properties

**DOI:** 10.3390/molecules30091934

**Published:** 2025-04-26

**Authors:** Bussagon Thongbai, Duljira Sukboonyasatit, Kriangsak Banlue, Sudathip Inchuen, Wanida Chuenta, Sirithon Siriamornpun, Sarinthorn Suwannarong

**Affiliations:** 1 Department of Food Technology and Nutrition, Faculty of Technology, Mahasarakham University, Kantarawichai, Maha Sarakham 44150, Thailand; bussagon.t@msu.ac.th (B.T.); duljira.s@msu.ac.th (D.S.); kriangsak.b@msu.ac.th (K.B.); sudathip.i@msu.ac.th (S.I.); wanida.ch@msu.ac.th (W.C.); sirithon.s@msu.ac.th (S.S.); 2 Research Unit of Thai Food Innovation (TFI), Department of Food Technology and Nutrition, Faculty of Technology, Mahasarakham University, Kantarawichai, Maha Sarakham 44150, Thailand

**Keywords:** *Coffea arabica* L., phenolic compounds, flavonoids, caffeic acid, apigenin, sugar addition, coffee husk by–product, sustainable development

## Abstract

This study aims to evaluate the effects of different cascara particle sizes and variations in the kombucha fermentation process on the bioactive compounds and antioxidant properties of cascara (*Coffea arabica* L.) kombucha. Cascara tea (CT), cascara tea with sugar (CS), and cascara kombucha (CK) were prepared using whole, coarsely ground, and finely ground cascara. A finer particle size enhanced color intensity and improved the extraction of bioactive compounds. CK prepared with finely ground cascara demonstrated the highest total phenolic content (TPC), total flavonoid content (TFC), and ferric reducing antioxidant power (FRAP). Fermentation influenced the profile of phenolic acids, leading to a decline in most compounds, except for vanillic acid in all CK samples, which increased during fermentation. Interestingly, apigenin levels increased, while quercetin levels decreased throughout fermentation. These findings highlight the role of fermentation, sugar addition, and particle size reduction in enhancing phenolic extraction and antioxidant potential in cascara-based beverages, particularly cascara kombucha.

## 1. Introduction

Recent studies highlight the potential application of these bioactive compounds in various food products, particularly beverages, to enhance antioxidant intake. Among these, kombucha—a fermented tea—has gained attention as a promising medium for cascara infusion, offering both probiotic benefits and improved bioavailability of bioactive compounds [1]. Kombucha is currently one of the most widely consumed low alcohol fermented beverages and represents the fastest–growing segment within the functional beverage market [2]. The global kombucha market was valued at USD 1.67 billion in 2019 and approximately USD 2.2 billion in 2020, with a projected revenue-based compound annual growth rate (CAGR) of around 20% through 2027 [3].

Coffee cherries, the fruit of the coffee plant, encase the coffee beans, which are the seeds found within the cherries. The dried outer layer of the coffee cherry, known as cascara, has garnered interest for its potential use as a functional ingredient, primarily due to its high concentration of bioactive compounds. Recent studies have highlighted cascara as a rich source of health–promoting substances, including antioxidants, phenolic compounds, and flavonoids [4,5]. Phenolic compounds, which are abundant in coffee cascara, exhibit strong antioxidant activity, playing a key role in mitigating oxidative stress and reducing the risk of chronic diseases. Flavonoids, a subclass of phenolics, also contribute significantly to antioxidant capacity and have been linked to various health benefits, including anti–inflammatory, anti–carcinogenic, and cardioprotective effects [6]. Notably, the fermentation process in kombucha has been shown to enhance the bioavailability of these compounds, as microbial activity can break down complex molecules into simpler, more readily absorbable forms [7].

When coffee cascara is used as a substrate for kombucha fermentation, it not only imparts a unique flavor profile but also has the potential to enhance the beverage’s nutritional value by increasing the concentration of health–promoting compounds. The antioxidant capacity of cascara–based kombucha can be significantly influenced by the particle size of cascara used in the fermentation process. Smaller particle sizes generally provide a greater surface area for extraction, potentially leading to higher concentrations of phenolic compounds and flavonoids in the final product [8,9]. However, excessively fine particles may reduce filtration efficiency and cause sedimentation issues. In plant materials containing starch or mucoproteins [10], particle disruption may also lead to increased viscosity or the formation of colloidal suspensions which can impede solvent penetration and reduce extraction efficiency [11]. Therefore, optimal particle size should balance improved surface area with practical extraction considerations. Despite its importance, the effect of particle size on the extraction efficiency and subsequent antioxidant activity in cascara kombucha remains underexplored. Additionally, the fermentation process, driven by a symbiotic culture of bacteria and yeast (SCOBY), can alter the chemical composition of cascara, potentially enhancing its antioxidant properties [12]. The interaction between cascara particle size and fermentation conditions presents an opportunity to optimize cascara kombucha formulations for enhanced health benefits.

This study aims to evaluate the effect of different cascara particle sizes and variations in the kombucha fermentation process on the antioxidant activity of cascara kombucha, focusing specifically on phenolic compounds, flavonoids, and other bioactive compounds. A control group—cascara tea with added sucrose (CS)—was included to assess the effects of sugar addition in the absence of fermentation. This comparison with the fermented cascara tea kombucha (CK) group allows for differentiation between the impacts of fermentation and sucrose alone. While sucrose is not expected to directly alter bioactive compound levels, it may affect compound solubility, extraction efficiency, or stability during beverage preparation. The findings of this study are expected to support the development of optimized cascara kombucha formulations with improved health benefits and consumer appeal.

## 2. Results and Discussion

### 2.1. Physical Properties

The study investigated the pH, total soluble solids (TSS), and color across of three preparation stages: cascara tea (CT), cascara tea with sugar (CS), and cascara kombucha (CK), each prepared with different particle sizes. The results are presented in Table 1.

The pH values of the nine tea samples ranged from 3.22 to 4.26, indicating variations in acidity levels among the different preparations. CT and CS samples exhibited higher pH values, ranging from 4.13 to 4.26, while CK samples showed lower pH values, ranging from 3.22 to 3.34 (*p* < 0.05). The reduction in particle size in each preparation stage was not statistically significant at the 95% confidence level. With CK samples demonstrating more acidic properties than CT and CS samples. The lower pH values in CK are attributed to the fermentation process, which produces organic acids, thereby increasing the acidity [13]. Previous studies have reported that the pH of cascara from various coffee varieties ranges from 2.69 to 5.3 and fermented 10–14 days [14,15]. This variation may be influenced by factors such as processing methods and coffee cultivar differences. According to The Centre of Disease Control of British Columbia (BCCDC) plan, pH levels should not be below 2.5 for the commercial kombucha–making process [16]. Generally, a pH below 4.6 is recommended for food safety, as it helps inhibit the growth of harmful bacteria [17]. The achieved pH in this study falls well within the microbiological safety range. Although glucuronic acid is a known fermentation product in kombucha, it was not quantified in this study due to the lack of an authentic standard. Nevertheless, its formation is well documented in the literature [12,18,19,20] and it is likely to be present in the cascara kombucha samples analyzed.

TSS values, expressed as percentages, indicating the dissolved solids at 20 °C are mainly sucrose sugar present in the fruit and may consist of organic acids, pigments, vitamins, and protein [21]. The TSS values of tea ranged from 0.50 to 11.15% are divided into three groups with significant differences. CT samples had the lowest TSS values, ranging from 0.50 to 0.60%, exhibiting their preparation method which involves minimal addition of soluble substances. In contrast, CS and CK samples showed significantly higher TSS values, ranging from 10.00 to 11.15%, with CK being slightly lower than CS. Moreover, the differences in particle size within each preparation stages did not significantly affect TSS concentrations (*p* ≥ 0.05), indicating that the incorporation of sugar and heating temperature in CS, and across fermentation process in CK contributed to generally increase soluble solid content. The addition of sugar increases the TSS content as simple sugars (mono– and disaccharides) are dissolved. However, during prolonged fermentation, microbes metabolize the sugar and convert into glucose, which serves as a nutrient for fermenting microbes [22]. Yeasts, such as *Saccharomyces cerevisiae*, ferment sugars into ethanol. Subsequently, acetic acid bacteria, like *Acetobacter xylinum*, oxidize this ethanol into acetic acid, decreasing the pH of the beverage [7,22]. As a result, microbial growth and activity lead to a decrease in total soluble solid content [22].

The color attributes of cascara tea (CT), cascara tea with sugar (CS), and cascara kombucha (CK) were significantly influenced by particle size and processing methods. Lightness (L*), redness (a*), and yellowness (b*) values provided key insights into the visual characteristics of these beverages (Table 1). L* values ranged from 27.48 to 28.11, indicating relatively dark–colored samples. CS and CK generally exhibited higher L* values, suggesting that sugar addition and fermentation contributed to a lighter appearance. Remarkably, CK3 (finely ground cascara kombucha) had the lowest L* value (27.48), implying that finer particle sizes enhanced pigment extraction, resulting in a darker hue.

The a* values (redness) varied from 0.33 to 0.70, with CT2 (4–6 mm) exhibiting the highest a* value (0.70), indicating greater retention of red pigments in non-fermented samples. Conversely, CK samples displayed the lowest a* values (0.33–0.34), likely due to anthocyanin degradation during fermentation. This decline in red tones can be attributed to the enzymatic activity of β–glycosidase produced by yeast, which hydrolyzes anthocyanins and alters the sensory and visual characteristics of kombucha [23]. CS samples exhibited intermediate a* values (0.48–0.57), suggesting that sugar addition may mitigate color degradation during processing. The b* values (yellowness) ranged from 6.24 to 7.05, with CT2 displaying the highest b* value (7.05), indicative of a more pronounced yellow hue. In contrast, CK samples exhibited the lowest b* values (6.24–6.26), potentially due to oxidative degradation of polyphenols and pigments during fermentation [24]. CS samples showed intermediate b* values (6.54–6.99), aligning with their moderate a* values, further supporting the role of sugar in color stabilization.

Fermentation-derived organic acids influenced color development by lowering pH and altering the chemical composition, resulting in darker shades. Smaller particle sizes enhanced pigment extraction, contributing to lower L* values and more intense coloration. Among non-fermented samples, CT2 (4–6 mm) exhibited the most vibrant hue, suggesting an optimal balance of pigment retention. The intermediate color characteristics of CS samples may stem from caramelization or Maillard reactions, producing melanoidins and other pigments [25]. Additionally, kombucha color variations may be attributed to phenolic biotransformation, where polymerization or depolymerization modifies pigment composition, either intensifying or diminishing color [9]. These findings highlight the pivotal role of particle size, sugar content, and fermentation in shaping cascara tea’s final color, which may influence consumer perception and acceptance.

### 2.2. TPC, TFC, and Antioxidant Activity in Cascara Kombucha Production

The bioactive compounds in cascara kombucha including total phenolic content (TPC) and total flavonoid content (TFC), as well as their bioactivity at various stages of kombucha production are shown in Table 2. The highest TPC was observed in CK3, followed by CS3, CK2, and CK1, indicating that the fermentation process further increased TPC levels. The addition of sugar and heat during preparation, prior to fermentation, significantly enhanced phenolic content. In contrast, CT exhibited lower TPC compared to both CS and CK due to the absence of sugar, heating, and fermentation, which results in lower phenolic content. Additionally, reducing the particle size of cascara improved phenolic extraction, as demonstrated by the higher TPC values in finely ground samples (CT3, CS3, CK3) compared to uncrushed or coarsely ground samples.

The increase in TPC in cascara kombucha (CK) samples can be attributed to microbial activity during fermentation. Microorganisms, primarily acetic acid bacteria and yeast, release various enzymes such as cellulase, glucanase, xylanase, pectinase, glucosidase, amylase, and invertase. These enzymes degrade the cell wall structures of small cascara fragments present in the extract, allowing them to pass through a filter cloth and releasing bound phenolic compounds from the plant matrix [26]. Additionally, they break down larger polyphenols and other compounds into smaller molecules [14], thereby increasing the TPC in CK. Moreover, enzymatic activity leads to the formation of phenolic compounds as a result of degradation processes [22]. This is consistent with studies demonstrating that fermentation enhances phenolic content in substrates such as coffee by-products and Pu–erh tea [13,14,27]. However, some studies have reported a reduction in bioactive compounds during prolonged fermentation. This decline may be due to the degradation of polyphenols by microbial enzymes or their consumption by microorganisms [28]. Moreover, the increase in TPC can be attributed to the acid hydrolysis process that occurs during the fermentation process. This mechanism has been supported by several studies. For example, Qin et al. [29] and König et al. [30] demonstrated that acid hydrolysis typically around pH 2.0 during fermentation can effectively release bound phenolics from complex substrates, leading to an increase in TPC. Similarly, Gaur and Gänzle [31] reported that the acidic conditions during fermentation facilitate the degradation of condensed tannins into monomeric phenolic acids, which substantially enhance the overall phenolic content and antioxidant capacity of the fermented product. In the present study, although the pH significantly decreased during fermentation (Table 1), it remained within an acidic range favorable for the breakdown of phenolic compounds, thereby contributing to the increased total phenolic content (TPC) observed in the final product.

The value of TFC was highest at different processing stages in CT3, followed by CS3 and CK3. However, CK1 and CK2 exhibited the lowest values. These findings suggest that reducing cascara particle size enhances flavonoid extraction, aligning with the trends observed for TPC. However, the lower TFC in CK samples compared to CT and CS may be attributed to the degradation or transformation of flavonoids by microbial enzymes released during fermentation, which may convert flavonoids into other bioactive compounds with potential health benefits. This phenomenon has been similarly reported in the fermentation of raw Pu–erh tea kombucha, where a significant increase in TPC and a prominent decrease in TFC were observed [13]. Furthermore, studies by Hsieh et al. [18] and Jakubczyk et al. [32] demonstrated that flavonoid degradation occurs during the fermentation of kombucha made from white, green, dark, and black teas.

Regarding antioxidant activity, as shown in Table 2, CS3 exhibited the highest DPPH radical scavenging activity, followed by CT3, CS2, and CS1, while CK2 displayed the lowest activity. These findings suggest that the addition of sugar and heating increases DPPH activity, which subsequently decreases after fermentation. It was noticed that CK3 demonstrated higher DPPH activity than CK2 and CK1. In contrast, CK3 had the highest ferric reducing antioxidant power (FRAP) value, followed by CK2 and CK1, with CT1 and CT2 showing the lowest values. This indicates that the combination of sugar addition, heating, and fermentation collectively enhances FRAP values. The antioxidant analysis revealed a strong association between DPPH activity and total flavonoid content (TFC), and between FRAP values and total phenolic content (TPC). The contrasting trends in DPPH and FRAP post–fermentation may be explained by their differing antioxidant mechanisms. Previous studies have shown that fermentation enhances kombucha’s reducing power and overall antioxidant capacity by increasing the content and bioavailability of phenolic compounds [15].

Furthermore, the content and composition of bioactive compounds in kombucha could be significantly influenced by several factors, including the variety of coffee, the specific coffee by–products used (such as husk or pulp), and the fermentation parameters. These parameters include the type and concentration of sugar, fermentation duration, temperature, as well as the specific microbial strains and their concentrations. Collectively, these variables play a critical role in shaping the composition and biological activity of the active ingredients present in kombucha [14,15,19,22,27,33]. Reducing cascara particle size significantly improved the extraction of bioactive compounds, resulting in higher TPC, TFC, DPPH, and FRAP values in finely ground samples (CT3, CS3, CK3). The smaller particle size increased the surface area of the cascara, allowing better solvent contact and more efficient extraction of phenolic and flavonoid compounds [34,35,36]. This phenomenon has been documented in food processing, where fine grinding disrupts cell walls and releases intracellular compounds that contribute to bioactivity. The increased extraction efficiency, combined with enhanced phenolic compound release during fermentation, likely accounts for the higher bioactivity observed in CK3 samples, emphasizing the importance of optimizing particle size for functional beverage production.

### 2.3. Phenolic Acid and Flavonoid Contents in Cascara Kombucha Production

The identification of phenolic compounds is shown in Table 3. There were 12 phenolic acids identified in the samples during kombucha production using HPLC, which can be classified into two groups: hydroxycinnamic acids and hydroxybenzoic acids. The hydroxycinnamic acids identified were chlorogenic acid, caffeic acid, *p*–coumaric acid, cinnamic acid, ferulic acid, and sinapic acid, while the hydroxybenzoic acids included hydroxybenzoic acid, gentisic acid, gallic acid, protocatechuic acid, syringic acid, and vanillic acid. 

Regarding the antioxidant structure, the –CH–CH–COOH in hydroxycinnamic acid provides better antioxidant activity than the –COOH in hydroxybenzoic acid. It is likely that the –CH–CH section gains structural resonance, helping to stabilize the free radicals [37]. Gallic acid has stronger antioxidant activity than hydroxybenzoic acid. The antioxidant activity of hydroxycinnamic acids is ranked in descending order as follows: ferulic acid > *p*–coumaric acid > sinapic acid > *o*–coumaric acid > *m*–coumaric acid > caffeic acid > chlorogenic acid [38]. Among these, the most abundant were *p*–hydroxybenzoic acid, followed by chlorogenic acid, gentisic acid, caffeic acid, gallic acid, and *p*-coumaric acid. Previous studies by Pua et al. [39] and Heeger et al. [40] also identified hydroxycinnamic acid, caffeoylquinic acid, chlorogenic acid, protocatechuic acid, and gallic acid in cascara samples. However, this research detected gentisic acid, which was not reported in earlier studies. Gentisic acid (GA, 2,5–dihydroxybenzoic acid) is a secondary metabolite of salicylic acid, known for its strong antioxidant and anti–inflammatory properties. In fermented blueberries, gentisic acid (96.90 ng/mL) has been reported to reduce inflammation and enhance the effectiveness of other bioactive compounds in inhibiting cancer cell proliferation and survival [41]. Our findings demonstrate that different stages of kombucha production distinctly influenced the phenolic acid profile. The addition of sugar and application of heat generally led to an increase in the levels of most phenolic acids. In contrast, fermentation resulted in a reduction of several phenolic acids, with the exception of gentisic and gallic acids in CK1, and vanillic acid across all CK samples, which increased during fermentation. A comparison between CT and CS samples revealed that CS generally contained higher concentrations of phenolic acids than CT, except for sinapic acid and gentisic acid, which were significantly lower in CS1 compared to CT1 (Table 3).

The heating process can break down cell walls and other structural components of the cascara, facilitating the release of bound phenolic acids. Heat also increases the solubility of phenolic acids, enhancing their extraction from the cascara matrix. The thermal energy provided by heating disrupts interactions between phenolic compounds and macromolecules such as proteins and polysaccharides, resulting in a higher concentration of free phenolic acids [42].

Changes in specific phenolic acids during the fermentation of cascara kombucha can be attributed to microbial metabolism and chemical transformations under acidic fermentation conditions [8]. The microbial community in kombucha, consisting of various bacterial and yeast strains, selectively degrades some phenolic acids while increasing the concentrations of others through enzymatic reactions. The acidic environment promotes hydrolysis, breaking down complex polyphenols into simpler phenolic acids [20]. The observed increase in gallic acid and vanillic acid during fermentation can be attributed to the hydrolysis of complex precursors, such as tannins and ferulic acid, as well as microbial metabolic processes that convert other phenolic compounds into these acids [43].

The effect of cascara particle size reduction on phenolic acid content was inconsistent. For example, size reduction did not significantly impact gallic acid levels during extraction, sugar addition, and heating, but unground cascara showed higher gallic acid levels post-fermentation compared to ground samples. Conversely, reducing cascara size increased caffeic and chlorogenic acid contents, while gentisic acid levels decreased in CT and CK samples. The changes in phenolic acid content during kombucha processing are influenced by their chemical properties, stability, and location within the plant matrix [40]. In unground samples, the intact structure protects bound gallic acid, allowing for gradual release during fermentation, whereas ground samples, with increased surface area, facilitate faster microbial degradation [44].

Additionally, Table 4 presents the six flavonoids identified in the samples. Apigenin and quercetin were the predominant flavonoids in cascara extract, accounting for 67.89% of the total flavonoid content. The remaining flavonoids included myricetin, kaempferol, and rutin, with catechin absent in all analyzed samples. According to Pua et al. [39] and Heeger et al. [40], flavonoids and xanthonoids were detected in cascara, but not catechin. This absence could be attributed to the degradation of catechin during processing and storage or the sample preparation technique used. Overall, fermentation significantly increased the total content of flavonoids, except in CT1 (Table 4). CT1 contained the highest levels of apigenin and kaempferol, but the lowest levels of quercetin and rutin. However, the addition of sugar and heating reduced apigenin levels, while quercetin and rutin levels increased. Kaempferol and myricetin showed only minor changes. During fermentation, apigenin levels increased, whereas quercetin levels decreased. Reducing cascara particle size enhanced the extraction of quercetin and rutin in the initial stages but lowered apigenin and kaempferol levels, affecting their concentrations in later stages of kombucha production.

The differential effects of sugar addition and heating on flavonoid levels in cascara extract can be attributed to the unique chemical properties of each compound. Apigenin is a bioflavonoid widely present in plant-based foods and is known for its biological activities, including protection against immune, cardiovascular, neurodegenerative diseases, and cancer [45]. Liu et al. [46] reported a significant decrease in apigenin levels following heat treatment at 37 °C and 100 °C or with Fe^2^^+^/Cu^2^^+^ supplementation, with the greatest reduction in apigenin’s bioactivity occurring under high temperatures. However, changes in apigenin content can vary depending on the treatment, food matrix, and processing conditions [45]. The observed changes in flavonoid contents during fermentation could be resulted from a balance between the degradation of certain compounds and the release or synthesis of others, influenced by the specific fermentation conditions and the microbial composition involved.

## 3. Materials and Methods

### 3.1. Materials and Chemicals

Cascara tea (*Coffea arabica* L. (family Rubiaceae)) was sourced from a local coffee plantation in Chiang Mai, Thailand. The dried cascara was ground using a blender (Panasonic MX–AC400, Bangkok, Thailand) for 5 s. The ground material was then sieved using a No. 20 mesh to separate two particle size fractions: finely ground particles (0.5–1.0 mm), which passed through the sieve, and coarsely ground particles (approximately 4–6 mm), which were retained on the sieve. Na_2_CO_3_, Folin–Ciocalteu, gallic acid, AlCl_3_, NaNO_2_, quercetin, 2, 2–diphenyl–1–picrylhydrazyl (DPPH) reagent, and ascorbic acid were purchased from Sigma–Aldrich Co. (St. Louis, MO, USA).

### 3.2. Preparation of Kombuchas

**Figure 1:**
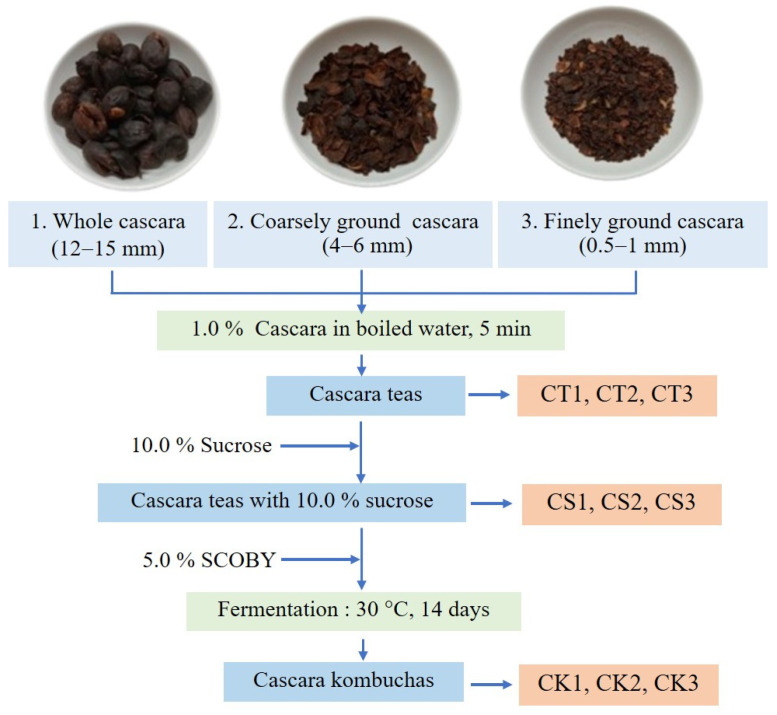
Cascara kombucha preparation.

Three different particle sizes of cascara tea were prepared: whole cascara (CT1), coarsely ground (4–6 mm, CT2), and finely ground (0.5–1 mm, CT3) (Figure 1). Each sample (10 g, 1.0%*w*/*w*) was immersed in 1.0 L of boiling water for 5 min. After removing the tea leaves, 100 g (10.0%*w*/*w*) of sucrose was added to the tea broth and stirred until fully dissolved (CS1, CS2, CS3). Once cooled, 50 g (5.0%*w*/*w*) of SCOBY (kombucha pellicle) was introduced into a bottle container covered with cheesecloth. Fermentation was conducted at room temperature for 14 days. The resulting cascara kombucha samples (CK1, CK2, CK3) were collected and stored at 5 °C for physicochemical analysis. All samples were fermented in three replications. The preparation methods for each treatment are illustrated in Figure 1.

### 3.3. Determination of pH and Total Soluble Solid

The pH values of cascara teas and kombuchas were measured using a digital pH meter [47]. Total soluble solids (TSS) in cascara teas and kombuchas were analyzed in a digital refractometer (0–53% Brix, PAL–1, ATAGO, Japan) and expressed in %Brix.

### 3.4. Measurement of Color

Color of cascara teas and kombuchas were determined using colorimeter (Konica Minolta, Chroma Meter CR–400, Tokyo, Japan). The instrument was calibrated with a standard white plate. The color parameters were L* (lightness), a* (redness), and b* (yellowness).

### 3.5. Determination of Total Phenolic Content

The quantification of total phenolic content (TPC) was assessed using Folin–Ciocalteu assay. Briefly, 0.5 mL of cascara teas and cascara kombuchas were mixed with 2.50 mL of Folin–Ciocalteu reagent (10%*v*/*v*). The mixtures were gently mixed and left to incubate at room temperature for 4 min. Subsequently, 7.5%*w*/*v* Na_2_CO_3_ (2 mL) was added, and the mixtures were mixed and incubated in the dark for 30 min at room temperature. The absorbances of the mixtures were measured at 760 nm using a spectrophotometer (DR2700, HACH, Germany). Results were expressed in micrograms of gallic acid equivalent (GAE) per mL of sample [48].

### 3.6. Determination of Total Flavonoid Content

Total flavonoid content (TFC) was evaluated using the modified AlCl_3_ colorimetric assay [49]. Briefly, 1 mL of cascara teas and cascara kombuchas were mixed with 0.3 mL of NaNO_2_ (5%*w*/*v*) kept for 5 min at room temperature. Then, 0.5 mL AlCl_3_ (2%*w*/*v*) was added, followed by another 6 min incubation at room temperature before 0.5 mL NaOH (1M) was added. The final mixture was stored for 10 min at room temperature and the absorbance was measured using a spectrophotometer at 510 nm. Quercetin was used as a standard to obtain the calibration curve (0–300 µg/mL). TFC expressed as microgram of quercetin equivalent per milliliter (µg QE/mL).

### 3.7. Radical Scavenging Activity by DPPH Assay

The scavenging activity of cascara teas and cascara kombuchas were evaluated according to the modified protocol described by Nopparat et al. [50]. In brief, tea and kombucha samples (0.5 mL) were mixed with 4.5 mL DPPH (0.06 mM) and left to incubate in the dark for 30 min at room temperature. The absorbances of the mixtures were measured at 517 nm by spectrophotometer. The antioxidant activity of the mixtures was expressed in microgram of ascorbic acid equivalent per milliliter (µg AE/mL).

### 3.8. Ferric–Reducing Antioxidant Power (FRAP) Assay

Determination of the antioxidant activity by ferric–reducing antioxidant power (FRAP) assay was determined. The FRAP reagent was freshly prepared by mixing 100 mL of acetate buffer (0.3 M, pH 3.6), 10 mL FeCl_3_.6H_2_O (20 mM), and 10 mL TPTZ solution (10 mM TPTZ in 40 mM HCl) in ratio of 10:1:1. Volumes of 4.5 mL of FRAP reagent and 0.1 mL of cascara teas and cascara kombuchas were mixed and incubated for 10 min in the dark. Absorbances were determined at 593 nm, using the FRAP working solution as blank. The data were expressed as microgram ferrous sulfate per milliliter (µg FeSO_4_/mL) [48].

### 3.9. HPLC Determination of Phenolic and Flavonoid Compounds

Phenolic acids and flavonoids were analyzed using the HPLC method. The condition of HPLC (series 20, Shimadzu, Kyoto, Japan) was as follows: C18 column (250 mm × 4.6 mm, 5 µm; InertSustain, GL Sciences, Tokyo, Japan). The mobile phase consisted of purified water with 1% (*v*/*v*) acetic acid (solvent A) and acetonitrile (solvent B) at a flow rate of 0.8 mL/min. A linear gradient elution was performed as follows: 0–5 min, 5–9%B; 5–15 min, 9%B; 15–22 min, 9–11%B; 22–38 min, 11–18%B; 38–43 min, 18–23%B; 43–44 min, 23–90%B; 44–45 min, 90–80%B; 45–55 min, 80%B; 55–60 min, 80–5%B. A re–equilibration period of 5 min with 5%B was used between individual runs. Operating conditions were as follows: column temperature, 38 °C; injection volume, 20 µL. The wavelengths for detecting hydroxybenzoic acids, hydroxycinnamic acids, and flavonoids were 280, 320, and 370 nm, respectively. The phenolics in the extracted samples were identified according to the retention time and spectrum of each standard [51].

### 3.10. Statistical Analysis

The experiments were conducted using completely randomized design (CRD). Data analyses were carried out by one-way analysis of variance. Differences among the mean values were compared by Duncan’s new multiple range test (*p* < 0.05). Data were collected in three replications for each sample and presented as the mean ± standard deviation (SD).

## 4. Conclusions

This study demonstrates that cascara particle size and fermentation processes significantly impact the physicochemical properties, bioactive compound content, and antioxidant activity of cascara kombucha. Fermentation notably increases acidity and influences the phenolic profile, enhancing antioxidant potential. The addition of sugar elevates total soluble solids (TSS) and, when combined with heating, improves bioactive compound extraction. Reducing cascara particle size further enhances the extraction efficiency of phenolic compounds and flavonoids. Among the studied samples, cascara kombucha prepared with finely ground cascara (CK3) exhibited the highest levels of bioactive compounds and antioxidant activity. These findings highlight the potential for optimizing particle size and fermentation conditions to develop cascara kombucha with enhanced functional and health-promoting properties.

## Figures and Tables

**Table 1:** pH, total soluble solids (TSS), and color parameters (L*, a*, b*) of cascara tea and kombucha samples.

Parameters	Cascara Tea and Kombucha Samples								
	CT1	CT2	CT3	CS1	CS2	CS3	CK1	CK2	CK3
**pH**	4.16 ± 0.16 ^a^	4.25 ± 0.13 ^a^	4.26 ± 0.16 ^a^	4.13 ± 0.25 ^a^	4.185 ± 0.28 ^a^	4.21 ± 0.28 ^a^	3.29 ± 0.06 ^b^	3.34 ± 0.11 ^b^	3.22 ± 0.03 ^b^
**TSS (%Brix)**	0.50 ± 0.14 ^c^	0.60 ± 0.00 ^c^	0.55 ± 0.07 ^c^	11.15 ± 0.49 ^a^	10.70 ± 0.57 ^ab^	10.55 ± 0.21 ^ab^	10.90 ± 0.14 ^ab^	10.15 ± 0.78 ^b^	10.00 ± 0.14 ^b^
**Color**									
***L*** *	27.80 ± 0.05 ^ab^	27.55 ± 0.04 ^b^	27.73 ± 0.09 ^ab^	28.11 ± 0.32 ^a^	28.10 ± 0.10 ^a^	28.00 ± 0.34 ^a^	28.10 ± 0.11 ^a^	28.11 ± 0.12 ^a^	27.48 ± 0.30 ^b^
***a*** *	0.63 ± 0.01 ^ab^	0.70 ± 0.04 ^a^	0.61 ± 0.02 ^ab^	0.48 ± 0.06 ^c^	0.48 ± 0.01 ^c^	0.57 ± 0.05 ^b^	0.34 ± 0.03 ^d^	0.33 ± 0.02 ^d^	0.62 ± 0.08 ^ab^
***b*** *	6.88 ± 0.07 ^abc^	7.05 ± 0.07 ^a^	6.79 ± 0.06 ^bc^	6.71 ± 0.13 ^cd^	6.54 ± 0.07 ^d^	6.99 ± 0.28 ^ab^	6.26 ± 0.06 ^e^	6.24 ± 0.09 ^e^	6.94 ± 0.16 ^abc^

Values are expressed as mean ± SD of triplicate measurements (n = 3). Different superscript letters (a–e) within the same row indicate significant differences at *p* < 0.05 among treatments, where CT = cascara tea, CS = cascara tea with sugar, CK = cascara kombucha, and numbers denote size reduction of cascara teas (1 = whole, 2 = 4–6 mm, 3 = 0.5–1 mm).

**Table 2:** TPC, TFC, DPPH, and FRAP values of cascara tea and kombucha samples.

Antioxidant Activity (µg/mL)	Cascara Tea and Kombucha Samples								
	CT1	CT2	CT3	CS1	CS2	CS3	CK1	CK2	CK3
**TPC**	338.42 ± 3.80 ^f^	329.30 ± 5.80 ^e^	372.98 ± 6.66 ^c^	366.49 ± 6.87 ^c^	348.60 ± 2.90 ^d^	392.63 ± 4.11 ^b^	385.44 ± 4.25 ^b^	383.51 ± 4.25 ^b^	424.04 ± 6.05 ^a^
**TFC**	320.40 ± 0.93 ^f^	350.89 ± 1.34 ^d^	432.49 ± 2.17 ^a^	334.47 ± 1.13 ^e^	369.53 ± 1.13 ^c^	426.32 ± 1.50 ^b^	216.57 ± 2.26 ^h^	215.33 ± 2.57 ^h^	240.27 ± 2.52 ^g^
**DPPH**	233.30 ± 1.73 ^d^	239.69 ± 1.90 ^c^	252.01 ± 0.95 ^b^	250.79 ± 0.46 ^b^	251.55 ± 0.70 ^b^	257.33 ± 0.95 ^a^	228.13 ± 2.06 ^e^	220.38 ± 2.34 ^f^	232.39 ± 3.04 ^d^
**FRAP**	554.88 ± 6.61 ^g^	561.13 ± 6.61 ^fg^	630.71 ± 9.38 ^d^	575.29 ± 6.29 ^f^	612.79 ± 7.64 ^e^	678.63 ± 9.92 ^c^	697.38 ± 13.23 ^b^	698.63 ± 8.20 ^b^	725.29 ± 5.05 ^a^

Values are expressed as mean ± SD of triplicate measurements (n = 3). Different superscript letters (a–h) within the same row indicate significant differences at *p* < 0.05 among treatments, where CT = cascara tea, CS = cascara tea with sugar, CK = cascara kombucha, and numbers denote size reduction of cascara teas (1 = whole, 2 = 4–6 mm, 3 = 0.5–1 mm).

**Table 3:** Phenolic acid content of cascara tea and kombucha samples.

Phenolic Acid Content (µg/mL)	Cascara Tea and Kombucha Samples								
	CT1	CT2	CT3	CS1	CS2	CS3	CK1	CK2	CK3
***Hydroxycinnamic acids***									
**Chlorogenic acid**	39.44 ± 0.23 ^g^	47.44 ± 0.39 ^c^	44.16 ± 0.09 ^d^	44.45 ± 0.35 ^d^	53.66 ± 0.43 ^a^	50.12 ± 0.19 ^b^	40.58 ± 0.28 ^f^	42.76 ± 0.21 ^e^	44.28 ± 0.13 ^d^
**Caffeic acid**	16.40 ± 0.06 ^f^	19.86 ± 0.17 ^c^	18.56 ± 0.07 ^d^	18.34 ± 0.13 ^d^	21.88 ± 0.75 ^a^	20.95 ± 0.17 ^b^	17.48 ± 0.66 ^e^	18.34 ± 0.45 ^d^	18.80 ± 0.03 ^d^
***p*** **-coumaric acid**	9.58 ± 0.82 ^bc^	8.67 ± 0.08 ^cd^	8.23 ± 0.02 ^d^	10.37 ± 0.63 ^ab^	10.61 ± 0.14 ^a^	8.95 ± 0.63 ^cd^	8.97 ± 0.34 ^cd^	7.18 ± 0.02 ^e^	8.63 ± 0.01 ^cd^
**Cinnamic acid**	6.99 ± 0.18 ^h^	9.51 ± 0.08 ^d^	10.08 ± 0.11 ^c^	9.34 ± 0.09 ^e^	11.11 ± 0.96 ^b^	11.88 ± 0.01 ^a^	6.70 ± 0.14 ^i^	7.41 ± 0.05 ^g^	8.27 ± 0.04 ^f^
**Ferulic acid**	4.83 ± 0.18 ^g^	7.38 ± 0.08 ^c^	6.49 ± 0.05 ^d^	5.94 ± 0.13 ^e^	8.36 ± 0.01 ^a^	8.09 ± 0.03 ^b^	4.35 ± 0.16 ^h^	5.17 ± 0.10 ^f^	5.69 ± 0.42 ^e^
**Sinapic acid**	0.17 ± 0.01 ^a^	0.15 ± 0.00 ^b^	0.15 ± 0.00 ^b^	0.15 ± 0.01 ^b^	0.15 ± 0.00 ^b^	0.15 ± 0.01 ^b^	0.14 ± 0.00 ^b^	0.15 ± 0.01 ^b^	0.14 ± 0.01 ^b^
***Hydroxybenzoic acids***									
***p*** **-hydroxybenzoic acid**	61.68 ± 0.22 ^c^	62.97 ± 0.45 ^b^	55.78 ± 0.12 ^e^	67.22 ± 0.64 ^a^	67.30 ± 0.36 ^a^	60.91 ± 0.36 ^d^	41.72 ± 0.16 ^f^	41.43 ± 0.10 ^f^	41.38 ± 0.12 ^f^
**Gentisic acid**	36.28 ± 3.35 ^a^	20.98 ± 1.55 ^h^	22.51 ± 0.55 ^g^	26.37 ± 0.51 ^f^	31.74 ± 1.82 ^c^	35.06 ± 2.43 ^b^	30.56 ± 0.91 ^de^	27.87 ± 0.99 ^ef^	28.09 ± 0.40 ^ef^
**Gallic acid**	10.62 ± 0.37 ^c^	10.38 ± 0.21 ^c^	10.53 ± 0.17 ^c^	12.89 ± 0.55 ^b^	12.44 ± 0.09 ^b^	12.40 ± 1.08 ^b^	15.08 ± 0.19 ^a^	8.49 ± 0.14 ^d^	10.02 ± 0.47 ^c^
**Protocatechuic acid**	1.02 ± 0.01 ^f^	1.13 ± 0.02 ^de^	1.23 ± 0.02 ^bc^	1.12 ± 0.03 ^de^	1.41 ± 0.05 ^a^	1.28 ± 0.04 ^b^	1.11 ± 0.06 ^e^	1.19 ± 0.09 ^cd^	1.20 ± 0.06 ^cd^
**Syringic acid**	0.13 ± 0.01 ^f^	0.24 ± 0.01 ^a^	0.18 ± 0.01 ^d^	0.19 ± 0.01 ^c^	0.23 ± 0.01 ^a^	0.21 ± 0.01 ^b^	0.11 ± 0.01 ^f^	0.11 ± 0.01 ^f^	0.15 ± 0.01 ^e^
**Vanillic acid**	0.38 ± 0.02 ^g^	0.45 ± 0.01 ^e^	0.42 ± 0.00 ^f^	0.47 ± 0.01 ^e^	0.52 ± 0.03 ^d^	0.53 ± 0.01 ^d^	0.65 ± 0.11 ^a^	0.61 ± 0.02 ^b^	0.58 ± 0.01 ^c^
***Total***	187.52 ± 3.79 ^d^	189.28 ± 0.59 ^d^	178.23 ± 0.85 ^e^	196.85 ± 1.48 ^c^	219.41 ± 0.81 ^a^	210.54 ± 2.62 ^b^	167.47 ± 0.67 ^f^	160.74 ± 1.05 ^g^	167.26 ± 1.78 ^f^

Values are expressed as mean ± SD of triplicate measurements (n = 3). Different superscript letters (a–h) within the same row indicate significant differences at *p* < 0.05 among treatments, where CT = cascara tea, CS = cascara tea with sugar, CK = cascara kombucha, and numbers denote size reduction of cascara teas (1 = whole, 2 = 4–6 mm, 3 = 0.5–1 mm).

**Table 4:** Flavonoid content of cascara tea and kombucha samples.

Flavonoid Content (µg/mL)	Cascara Tea and Kombucha Samples								
	CT1	CT2	CT3	CS1	CS2	CS3	CK1	CK2	CK3
**Apigenin**	4.32 ± 0.28 ^a^	2.01 ± 0.04 ^e^	2.06 ± 0.07 ^e^	2.40 ± 0.02 ^d^	1.72 ± 0.04 ^f^	1.68 ± 0.07 ^f^	3.69 ± 0.12 ^b^	3.44 ± 0.07 ^c^	3.43 ± 0.15 ^c^
**Catechin**	<LOQ	<LOQ	<LOQ	<LOQ	<LOQ	<LOQ	<LOQ	<LOQ	<LOQ
**Kaempferol**	1.29 ± 0.02 ^a^	1.08 ± 0.03 ^bc^	1.12 ± 0.04 ^b^	1.09 ± 0.01 ^bc^	1.07 ± 0.02 ^c^	1.08 ± 0.03 ^bc^	1.07 ± 0.01 ^c^	1.05 ± 0.01 ^c^	1.05 ± 0.01 ^c^
**Myricetin**	1.73 ± 0.13 ^c^	1.80 ± 0.06 ^c^	1.83 ± 0.01 ^bc^	1.88 ± 0.08 ^abc^	1.83 ± 0.05 ^bc^	1.81 ± 0.01 ^c^	1.98 ± 0.13 ^ab^	2.03 ± 0.03 ^a^	1.90 ± 0.14 ^abc^
**Quercetin**	4.18 ± 0.11 ^f^	4.42 ± 0.08 ^e^	4.73 ± 0.08 ^c^	4.75 ± 0.13 ^c^	5.49 ± 0.04 ^a^	5.53 ± 0.07 ^a^	4.56 ± 0.06 ^d^	4.79 ± 0.03 ^c^	4.95 ± 0.04 ^b^
**Rutin**	1.00 ± 0.03 ^e^	1.34 ± 0.05 ^c^	1.32 ± 0.01 ^c^	1.20 ± 0.05 ^d^	1.58 ± 0.03 ^a^	1.46 ± 0.05 ^b^	1.35 ± 0.05 ^c^	1.43 ± 0.01 ^b^	1.47 ± 0.05 ^b^
***Total***	12.52 ± 0.29 ^a^	10.65 ± 0.10 ^e^	11.08 ± 0.15 ^d^	11.32 ± 0.23 ^cd^	11.68 ± 0.09 ^b^	11.56 ± 0.13 ^bc^	12.65 ± 0.32 ^a^	12.74 ± 0.05 ^a^	12.80 ± 0.20 ^a^

Values are expressed as mean ± SD of triplicate measurements (n = 3). Different superscript letters (a–f) within the same row indicate significant differences at *p* < 0.05 among treatments, where CT = cascara tea, CS = cascara tea with sugar, CK = cascara kombucha, and numbers denote size reduction of cascara teas (1 = whole, 2 = 4–6 mm, 3 = 0.5–1 mm). <LOQ—below limit of quantification.

## Data Availability

Additional data are available on request.
